# Transcriptome profiles of hypothalamus and adrenal gland linked to haplotype related to coping behavior in pigs

**DOI:** 10.1038/s41598-019-49521-2

**Published:** 2019-09-10

**Authors:** Kevin Gley, Eduard Murani, Nares Trakooljul, Manuela Zebunke, Birger Puppe, Klaus Wimmers, Siriluck Ponsuksili

**Affiliations:** 1 Leibniz Institute for Farm Animal Biology (FBN), Institute for Genome Biology, Wilhelm-Stahl-Allee 2, D-18196 Dummerstorf, Germany; 2 Leibniz Institute for Farm Animal Biology (FBN), Institute of Genetics and Biometry, Wilhelm-Stahl-Allee 2, D-18196 Dummerstorf, Germany; 3 Leibniz Institute for Farm Animal Biology (FBN), Institute for Behavioral Physiology, Wilhelm-Stahl-Allee 2, 18196 Dummerstorf, Germany

**Keywords:** Biological sciences, Behavioural genetics, Transcriptomics

## Abstract

The hypothalamic-pituitary-adrenal (HPA) axis is an important component of neuroendocrine stress regulation and coping behavior. Transcriptome profiles of the hypothalamus and adrenal gland were assessed to identify molecular pathways and candidate genes for coping behavior in pigs. Ten each of high- (HR) and low- (LR) reactive pigs (n = 20) were selected for expression profiling based haplotype information of a prominent QTL-region on SSC12 discovered in our previous genome-wide association study (GWAS) on coping behavior. Comparing the HR and LR pigs showed 692 differentially expressed genes (DEGs) in the adrenal gland and 853 DEGs in the hypothalamus, respectively. Interestingly, 47% (17 out of 36) of DEGs found in both tissues were located in GWAS regions identified on SSC12, indicating that there are significant functional positional candidate genes for coping behaviour. Pathway analysis assigned DEGs to glucocorticoid receptor signaling in the adrenal gland. Furthermore, oxidative phosphorylation, mitochondrial dysfunction, and NGF signaling as well as cholecystokinin/Gastrin-mediated were identified in the hypothalamus. We narrowed the list of candidate genes in GWAS regions by analyzing their DEGs in the HPA axis. The top identified transcripts, including *ATP1B2*, *AURKB*, *MPDU1* and *NDEL1* provide evidence for molecular correlates of coping behavior in GWAS regions.

## Introduction

Psychosocial stress, as an inherent issue in modern (intensive) pig production, is a major concern for animal health and welfare. Chronic stress can result in decreases in growth rate, meat quality, and immune competence, with a consequential serious risk for opportunistic diseases^1,2^. To maintain homeostasis under stressful conditions, an individual pig undergoes coping, a biological process that involves physiological changes as well as behavioral responses. Inter-individual differences in coping capacity within a species have been observed in a broad variety of medical, psychological, and animal studies. Attributing factors include genetics, developmental stage, early lifetime experience, social support, etc.^3^. The hypothalamic-pituitary-adrenal axis (HPA axis), a major neuroendocrine system, comprises a complex interplay of direct influences and feedback interactions by the three endocrine glands to orchestrate critical body functions such as digestion, immunity, mood and emotions, sexuality, energy balance, and most importantly, the controlled reaction to stress^4^. The adrenal gland plays a crucial role by secreting stress hormones, i.e., glucocorticoids - primarily cortisol - from the adrenal cortex and catecholamines from adrenal medulla controlled by the HPA axis and sympathetic nervous system, respectively^5^. We therefore selected hypothalamic and adrenal gland tissue samples for whole transcriptome analysis.

Recent studies have classified stress response patterns as proactive (or active) type, also known as the classical fight-or-flight response which is behaviorally featured by territorial control and aggression, or as the conservation-withdrawal response type, characterized by immobility and a decreased level of aggression^3,6,7^. The physiological and neuroendocrine aspects of the proactive type include low hypothalamic-pituitary-adrenal (HPA) axis activity (basal) and reactivity (stressed) and high sympathetic and low parasympathetic reactivity accompanied by high testosterone activity. The reactive coping type exhibits normal basal HPA axis activity that increases under stressed conditions with low sympathetic reactivity and testosterone activity while parasympathetic reactivity is high^8^.

High- and low-resistance coping styles in pigs are commonly differentiated using the backtest, a behavioral test conducted by putting the pigs on their back on a V-shaped support for 1 min. High-resisting pigs attempt to escape more frequently and have a higher heart rate than low-resisting pigs^9^. Coping behavior correlates with various physiological characteristics^10,11^. Generally, low-resistant pigs with high HPA-axis activity and high parasympathetic activity are classified as reactive pattern, whereas high-resistant pigs with low HPA axis activity and high sympathetic activity are classified as proactive pattern^12,13^.

Among well-established porcine behavioral tests such as the open-field test, lesion scoring, and human approach test, the backtest is recognized as a heritable and repeatable measure and a good indicator for assessing coping style in pigs. Heredity is considered an important factor that influences behavioral patterns of pigs^14,15^. The heritability of backtest traits is reported to vary from 0.10 to 0.5 depending on the sample size and/or test conditions^16–19^. A recent report estimated backtest-trait heritability to be 0.37–0.56 which is quite high for behavioral traits^20^. In addition to heritability, a previous publication reported moderate intra-situational consistency and repeatability of the backtest across four test repetitions^19^. Moreover, a recent study investigating inter-situational consistency detected a clear relationship between the backtest and parameters of other porcine behavioral tests (e.g., the human approach test and open field test)^21^, underpinning distinguishable coping predispositions as well as the validity of the backtest. To our knowledge, the genome wide association study (GWAS) approach followed by functional analysis and molecular characterization of stress-response behavioral traits in pigs has not yet been thoroughly explored.

Our study aims to identify molecular pathways and candidate genes regulating coping behavior in pigs. Previously, our GWAS in German Landrace pigs (n = 294) identified several coping-behavior associated SNPs on *Sus Scrofa* chromosome (SSC) 12 in the region between 55 and 56 Mb^22^. Based on the haplotype information and coping behavior, a total of 20 pigs showing high reactive (HR, n = 10) and low reactive (LR, n = 10) coping behaviors were selected for transcriptome profiling of hypothalamus and adrenal gland tissues. Differentially expressed genes (DEGs) in the HR and LR groups were detected and important biological pathways were identified, linking genetics to coping behavior in pigs.

## Results

Pigs were selected for transcriptome profiling of hypothalamus and adrenal gland tissue based on the backtest haplotype and phenotype. We first calculated the haplotype association, testing the markers ALGA0066975, ALGA0121951, ASGA0055092, ASGA0105202, H3GA0034753, and MARC0073387 in all 294 individuals. All candidate markers were located on SSC12 position 55–56 Mb (Ensembl_Sscrofa_10.2) and were previously reported as single markers associated with backtest traits^22^. In total, 4 haplotype variants of 294 piglets were used to test the association against the backtest phenotype. Haplotype 1 (A/G/A/C/A/A, n = 180) and 2 (G/A/G/A/G/C, n = 14) were found to be associated with backtest traits (Table 1). Based on this information together with backtest parameters, animals were divided into two groups, the high reactive (HR) group and low reactive (LR) group, each including 10 animals. A summary of phenotypic data such as the backtest characteristics of latency, duration and frequency, and cortisol levels is shown in Table 2. HR and LR pigs clearly showed significant differences in the backtest parameters for latency (*p* = 0.0002), duration (*p* < 0.0001) and frequency (*p* < 0.0001). Latency, a measure of time elapsed to first struggle, was nearly five times higher in the LR than the HR pigs. In contrast, cumulative struggling duration of the HR pigs was about four times higher than that of the LR group. The HR pigs showed a higher frequency of struggling attempts than the LR pigs during the 1-minute testing period. Cortisol levels did not differ significantly between the LR and HR pigs.

**Table 1:** Statistical testing of association between 4 haplotype variants (H1-H4) of 294 piglets against backtest parameters duration, frequency and latency.

Dependent	Haplotype	Hapname	Window	F-Statistic	Prob > F
Duration	A_G_A_C_A_A	H1	1	10.246	0.00154
Duration	G_A_G_A_G_C	H2	1	9.667	0.00208
Duration	A_G_A_A_G_C	H3	1	1.930	0.16591
Duration	Rare	H4	1	1.217	0.27088
Frequency	A_G_A_C_A_A	H1	1	18.693	0.00002
Frequency	G_A_G_A_G_C	H2	1	15.535	0.00010
Frequency	A_G_A_A_G_C	H3	1	4.562	0.03360
Frequency	Rare	H4	1	1.440	0.23113
Latency	A_G_A_C_A_A	H1	1	13.945	0.00023
Latency	G_A_G_A_G_C	H2	1	7.930	0.00522
Latency	A_G_A_A_G_C	H3	1	5.281	0.02233
Latency	Rare	H4	1	0.582	0.44602

Data set lists F-statistics and associated probabilities (Prob > F) for each of the estimated haplotypes. Rare haplotypes are defined as haplotypes with a frequency lower than 0.05.

**Table 2:** Comparison of phenotype data between HR (high reactive) and LR (low reactive) group.

Parameter	HR group (n = 10)	LR group (n = 10)	*p-value* (behavior)
***Backtest Behavior***			
Latency	29.6 ± 3.8	154.6 ± 22.5	0.0002
Duration	118.9 ± 7.7	30.9 ± 8.9	<0.0001
Frequency	16.5 ± 0.5	4.8 ± 1.2	<0.0001
***Lab Assays***			
Cortisol (ng/ml)	76.5 ± 10.5	94.1 ± 11.2	0.2811

*P*-values were shown to be significant between HR and LR groups. Latency describes the lapse of time to first struggle, duration is the cumulative struggling time within 1-minute testing period and frequency defines the count of struggling attempts. All data are presented as mean ± s.e.m.

### Differentially expressed genes and pathway analysis

#### Hypothalamus

From a total of 47 880 probe sets on the snowball array, 17 351 passed filtering steps and were ready for further analysis. Mixed model analysis carried out in JMP Genomics 7 with sex and coping style as fixed effects and sire as a random effect revealed 853 differentially expressed (*p* < 0.05) genes between the HR and LR groups (Supplementary Table S1). The DEGs were further analyzed for their functional roles and biological pathways using Ingenuity Pathway Analysis (IPA). Five hundred forty-seven out of 853 DEGs were annotated in the IPA database. Two hundred seventy-four and 273 DEGs were up- and down- regulated, respectively, in HR pigs compared to LR pigs. Absolute fold changes (FC) of higher than 1.5 (FC < −1.5, FC > 1.5) were observed in 109 out of 853 DEGs (12.78%). *MOGAT2*, a gene involved in the synthesis of diacylglycerol and triacylglycerol from monoacylglycerol, was the most down-regulated of all IPA-identified DEGs in the HR group (FC = −1.83, *p = *0.0015), followed by *OAS1*, *MUC1*, *COX7A1*, and *FAH* with slightly smaller fold-changes. *GBP7* was the most up-regulated gene (FC = 2.25, *p* < 0.0001), followed by *NOP58*, *CXCL9*, *CALCRL*, and *PDIK1L* (Table 3). Top canonical pathways and their associated genes are displayed in Table 4. The results revealed that DEGs were most significantly associated with the oxidative phosphorylation pathway (9 upregulated, 2 downregulated) and mitochondrial dysfunction (11 upregulated, 3 downregulated). Additionally, 14-3-3-mediated signaling, NGF, SAPK/JNK, and cholecystokinin-gastrin-mediated signaling pathways were predicted to be activated, whereas Wnt/ß-Catenin and PEDF signaling pathways as well as NF-κB activation by viruses were inactivated based on the IPA knowledgebase.

**Table 3:** Top ten differentially expressed genes in hypothalamus based on fold changes (FC) of the HR in comparison to the LR group (HR/LR).

Probe Sets	Gene Symbol	Description	*p*-value	FC
037185	*MOGAT2*	monoacylglycerol O-acyltransferase 2	0.0015	−1.83
024354	*OAS1*	2′,5′-oligoadenylate synthetase 1, 40/46 kDa	0.0248	−1.57
005838	*MUC1*	mucin 1, cell surface associated	0.0014	−1.54
022465	*COX7A1*	cytochrome c oxidase subunit VIIa polypeptide 1	0.0402	−1.49
010733	*FAH*	fumarylacetoacetate hydrolase (fumarylacetoacetase)	0.0203	−1.49
035464	*PDIK1L*	PDLIM1 interacting kinase 1 like	0.0283	1.94
035843	*CALCRL*	calcitonin receptor-like	0.0174	1.99
011576	*CXCL9*	chemokine (C-X-C motif) ligand 9	0.0175	2.05
026960	*NOP58*	NOP58 ribonucleoprotein homolog (yeast)	0.0317	2.10
006185	*GBP7*	Guanylate Binding Protein 7	<0.001	2.25

**Table 4:** Top canonical pathways of upregulated (HR > LR) and downregulated (HR < LR) genes in hypothalamus tissue.

IPA Canonical pathway	Differential expression	*p*-value	No. of molecules	Associated genes
Oxidative Phosphorylation	HR > LR	< 0.001	11	*MT-CO1*, *MT-CO2*, *MT-CO3*, *MT-ND1*, *MT-ND5*, *MT-CYB*, *MT-ND3*, *MT-ND2*, *ATP5E*, *ATP5G2*, *COX7A1*
Mitochondrial Dysfunction	HR > LR	< 0.001	14	*ACO1*, *ATP5G2*, *COX7A1*, *MT-CO1*, *MT-CO2*, *ATP5E*, *MT-ND1*, *MT-ND5*, *MT-CYB*, *MAPK10*, *MT-CO3*, *MT-ND3*, *MT-ND2*, *PDHA1*
14-3-3-mediated Signaling	HR > LR	0.0053	9	*AKT2*, *ATM*, *CBL*, *KRAS*, *MAP3K5*, *MAPK10*, *PLCB1*, *TUBB2B*, *TUBB4B*
NGF Signaling	HR > LR	0.0090	8	*AKT2*, *ROCK2*, *MAPK10*, *RPS6KA3*, *KRAS*, *MAP3K5*, *ATM*, *TP53*
Cholecystokinin/Gastrin-mediated Signaling	HR > LR	0.0132	7	*CCK*, *GNA12*, *KRAS*, *MAPK10*, *PLCB1*, *ROCK2*, *SRF*
SAPK/JNK Signaling	HR > LR	0.0153	7	*ATM*, *DAXX*, *GNA12*, *KRAS*, *MAP3K5*, *MAPK10*, *TP53*
Wnt/ß-Catenin Signaling	HR < LR	0.001	10	*ACVR1*, *AKT2*, *BMPR2*, *CSNK1G1*, *FZD3*, *NR5A2*, *SOX11*, *TP53*, *WNT2B*, *WNT8B*
PEDF Signaling	HR < LR	0.0184	6	*AKT2*, *ATM*, *KRAS*, *ROCK2*, *SRF*, *TP53*
NF-κB Activation	HR < LR	0.0204	6	*AKT2*, *ATM*, *CCR5*, *EIF2AK2*, *ITGB2*, *KRAS*

#### Adrenal gland

In the adrenal gland, 19887 out of 47880 probe sets remained after filtering for downstream analysis. A mixed-model analysis revealed 692 DEGs between the two groups at *p* < 0.05 (Supplementary Table S2). Of these, 512 DEGs were annotated by IPA. Three hundred sixty-three DEGs were down-regulated in the HR compared to LR pigs. Twenty-three DEGs exhibited an absolute fold change greater than 1.5. The top five DEGs with increased mRNA transcript abundance in HR compared to LR pigs were *IGSF5*, *MLKL*, *EDRF1*, *MGAM*, and *PCDH7*. The top five genes with lower expression levels in the HR pigs were *ATP1B2*, *OAS1*, *PTGFRN*, *PRLR* and *SEPW1* (Table 5). The ATPase Na^+^/K^+^ transporting subunit beta 2 (encoded by *ATP1B2*) showed the highest absolute fold change (FC = −4.04, *p* < 0.0001) with expression levels in HR pigs lower than levels in LR pigs. Up-regulated (HR > LR) genes were mainly associated with PPARα/RXRα activation, whereas down-regulated (HR < LR) genes were related to EIF2 signaling, and glucocorticoid receptor signaling, estrogen receptor signaling (Table 6).

**Table 5:** Top ten differentially expressed genes in adrenal gland tissue based on fold changes (FC) of the HR in comparison to the LR group (HR/LR).

Probe Sets	Gene Symbol	Description	*p*-value	FC
302714	*ATP1B2*	ATPase, Na+/K+ transporting, beta 2 polypeptide	<0.0001	−4.04
311356	*OAS1*	2′-5′-oligoadenylate synthetase 1	0.025	−2.07
294501	*PTGFRN*	prostaglandin F2 receptor negative regulator	0.031	−1.79
325868	*PRLR*	prolactin receptor	0.024	−1.79
308475	*SEPW1*	selenoprotein W 1	0.002	−1.69
299303	*PCDH7*	protocadherin 7	0.042	1.57
321953	*MGAM*	maltase-glucoamylase	0.039	1.65
311904	*EDRF1*	erythroid differentiation regulatory factor 1	0.006	1.70
296057	*MLKL*	mixed lineage kinase domain-like	0.006	1.71
303826	*IGSF5*	immunoglobulin superfamily member 5	0.011	1.83

**Table 6:** Top canonical pathways of upregulated (HR > LR) and downregulated (HR < LR) genes in adrenal gland tissue of HR group predicted by IPA.

IPA Canonical pathway	Differential expression	*p-value*	No. of molecules	Associated genes
PPARα/RXRα Activation	HR > LR	0.0059	5	*IL1RAP*, *IRS1*, *PRKAB2*, *PRKCB*, *SMAD3*
EIF2 Signaling	HR < LR	0.0058	8	*EIF3F*, *PPP1R15A*, *RPL19*, *RPL28*, *RPL39*, *RPLP1*, *RPS26*, *RPSA*
Glucocorticoid Receptor Signaling	HR < LR	0.0078	10	*BAG1*, *BCL2L1*, *CD247*, *ELK1*, *NFKBIA*, *POLR2G*, *POLR2H*, *SMAD2*, *STAT5B*, *TAF3*
Estrogen Receptor Signaling	HR < LR	0.0106	6	*MED27*, *POLR2G*, *POLR2H*, *SPEN*, *SRC*, *TAF3*

Tissue-wide analysis of differentially expressed genes of both tissues in GWAS regions: In order to identify candidate genes in GWAS regions of both tissues, mixed-model analysis with yielded 1428 differentially expressed genes between the LR and the HR group in the tissue-wide analysis of both hypothalamus and adrenal gland. To functionally characterize the genetics of coping behavior and identify candidate genes, we overlaid these identified DEGs with our previous GWAS results^22^, as shown in Table 7. A total of 36 out of 1428 DEGs were found in proximity (50 kb upstream and downstream) of trait-associated SNPs. The major share (47.22%) was mapped especially on SSC12. The following 17 DEGs were closely located to the following SNPs: *LOC100737651* (ALGA0110005), *ACSF2* (ALGA0110005), *WSCD1* (SNP: ALGA0120076), *PLD2* (SNP: ASGA0105522), *PSMB6* (SNP: ASGA0105522), *VMO1* (SNP: ASGA0105522), *BCL6B* (SNPs: ALGA0066930, H3GA0034704), *LOC100519213* (SNP: ALGA0066945), *EIF4A1* (SNP: ASGA0055076), *MPDU1* (SNP: ASGA0055076), *ATP1B2* (SNP: ASGA0055076), *DNAH2* (SNP: M1GA0016958), *TRAPPC1* (SNPs: ASGA0102838, ALGA0066969), *ALOXE3* (SNP: M1GA0016964), *BORCS6* (SNP: ASGA0055092), *AURKB* (SNP: ASGA0055092) and *NDEL1* (SNP: ALGA0066986). The SNPs proximal to *DNAH2*, *TRAPPC1*, *ALOXE3* and *NDEL1* were associated with all three backtest traits (latency, duration and frequency) in our previous study^22^. The most prominent genes were *ATP1B2*, *MPDU1* and *AURKB*, showing highly significant differential expression (FDR < 0.0001) between the LR and the HR group. Chromosome 5 harbored the second largest proportion of DEGs (16.67%), followed by SSC6 (11.11%) and SSC17 (8.33%).

**Table 7:** Significantly (FDR < 0.05) differentially expressed genes in hypothalamus and adrenal gland located in genome wide association study (GWAS) regions.

SSC (Mb)^*^	Gene	*p-value*	FDR	FC**	SNP ID	Related trai^t***^
1 (123.2)	*FBN1*	0.003	0.009	1.48	INRA0004039	Dd12, tD
2 (61.3)	*KLF2*	0.006	0.021	−1.14	ALGA0013926	Fd5
4 (14.3)	*TRIB1*	0.002	0.008	−1.14	ASGA0018519	Dd19, Dd26
5 (8.1)	*TNRC6B*	0.009	0.030	1.22	H3GA0015326	Dd19, Dd26
5 (8.7)	*ATF4*	0.016	0.048	−1.11	ASGA0024056	Fd19, tF
5 (10.0)	*ANKRD54*	0.015	0.047	−1.16	INRA0018313	Dd26
5 (30.7)	*HELB*	0.014	0.044	1.36	ALGA0031539	Ld12, Fd12
5 (71.3)	*SLC2A13*	0.015	0.047	1.38	INRA0019895	Fd5
6 (17.4)	*NFAT5*	0.002	0.009	1.30	DIAS0001800	Fd12
6 (26.8)	*FTL*	0.011	0.036	−1.12	ALGA0120313	Fd19
6 (65.3)	*LRRC47*	0.010	0.032	−1.15	MARC0054213	Fd12
6 (74.7)	*EFHD2*	0.004	0.013	−1.22	H3GA0018181	Dd19
7 (49.1)	*FAH*	0.003	0.010	−1.40	ALGA0041790	Dd12
8 (136.6)	*RASGEF1B*	0.011	0.034	1.25	ALGA0050206	Ld12, Fd12
12 (26.7)	*LOC100737651*	0.001	0.003	−1.23	ALGA0110005	Fd26
12 (26.8)	*ACSF2*	0.011	0.035	−1.19	ALGA0110005	Fd26
12 (52.1)	*PLD2*	0.010	0.033	−1.19	ASGA0105522	Dd26
12 (52.1)	*PSMB6*	0.010	0.033	−1.19	ASGA0105522	Dd26
12 (52.1)	*VMO1*	0.005	0.016	−1.18	ALGA0066930	Ld12, Fd19
					H3GA0034704	Ld12, Fd19
12 (51.2)	*WSCD1*	0.010	0.032	−1.17	ALGA0120076	Dd26
12 (52.4)	*BCL6B*	0.005	0.016	−1.18	ALGA0066930	Ld12, Fd19
					H3GA0034704	Ld12, Fd19
12 (52.7)	*LOC100519213*	0.003	0.010	−1.18	ALGA0066945	Dd12, Dd19, Dd26, Fd12, Fd19, Fd26, tF, tD
12 (52.9)	*EIF4A1*,*CD68*	0.012	0.038	−1.10	ASGA0055076	Dd26, Fd26
12 (52.9)	*MPDU1*	<0.0001	<0.0001	−1.50	ASGA0055076	Dd26, Fd26
12 (52.9)	*ATP1B2*	<0.0001	<0.0001	−2.15	ASGA0055076	Dd26, Fd26
12 (53.0)	*DNAH2*	0.008	0.026	−1.16	M1GA0016958	Ld12, Dd12, Dd19, Dd26, Fd12, Fd19, Fd26, tL, tF, tD
12 (53.2)	*TRAPPC1*	0.007	0.022	−1.22	ASGA0102838	Dd12, Dd19, Dd26, Fd12, tL, tF, tD
					ALGA0066969	Ld12, Dd12, Dd19, Dd26, Fd12, Fd19, Fd26, tL, tF, tD
12 (53.3)	*ALOXE3*	0.0001	0.0006	−1.22	M1GA0016964	Ld12, Dd5, Dd19, Dd26, Fd5, Fd12, Fd19, Fd26, tL, tF
12 (53.4)	*BORCS6*	0.003	0.011	−1.20	ASGA0055092	Ld12, Dd5
12 (53.4)	*AURKB*	<0.0001	<0.0001	−1.17	ASGA0055092	Ld12, Dd5
12 (53.6)	*NDEL1*	0.005	0.017	1.23	ALGA0066986	Ld12, Dd5, Dd12, Dd19, Dd26, Fd12, Fd26, tF, tD
					ALGA0066981	Ld12, Fd5, Fd12, t
13 (23.3)	*ACVR2B*	0.008	0.027	1.10	DIAS0001116	Dd5
17 (28.4)	*INSM1*	0.016	0.049	−1.18	ASGA0076061	Dd5, Dd12, Fd5, Fd12, tF
17 (46.7)	*GDAP1L1*	0.007	0.023	−1.15	ALGA0095422	Fd5, tF
17 (56.9)	*C17H20orf108*	0.014	0.042	1.14	ALGA0096195	Ld12, Dd5, DD19, Dd26, Fd5, Fd12, Fd26, tF, tD
18 (50.6)	*OGDH*	0.005	0.001	−1.14	H3GA0051149	Dd26, Fd19, Fd26

^*^Annotation based Ensembl_Sscrofa_11.1.

^**^FC = fold changes of the HR in comparison to the LR group (HR/LR).

^***^Related traits: L = Latency, the time elapsed until the first struggling attempt; D = Duration, the cumulative struggling time within the 60 s test; F = frequency, the number of struggling attempts within the 60 s test; tL = total latency, tD = total duration, and tF = total frequency which were calculated for each animal by summing the relevant parameters at all ages tested. The age of measures was labeled as d5, d12, d19 and d26.

**Figure 1:**
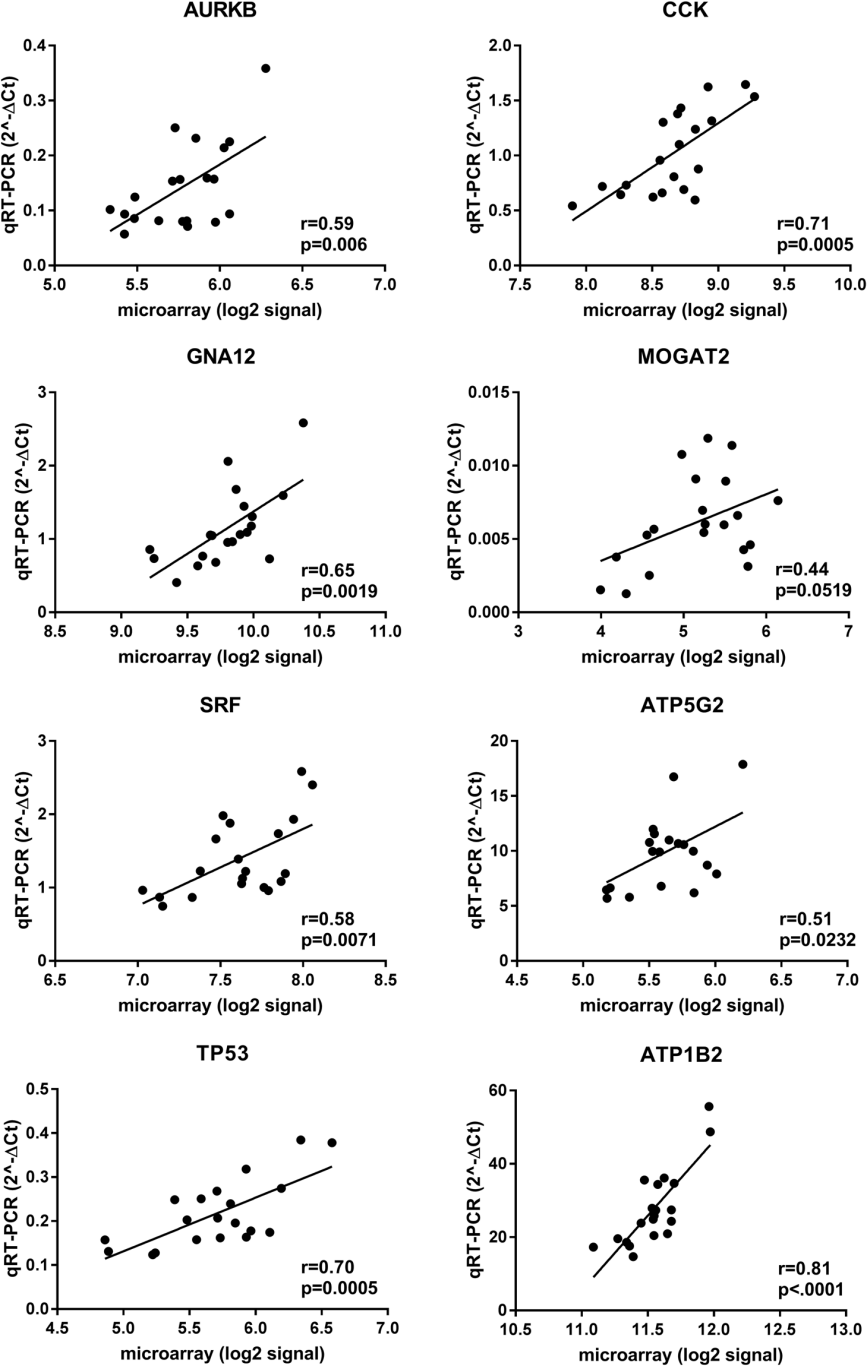
qPCR validation of selected genes of the hypothalamus microarray study, including *AURKB*, *CCK*, *GNA12*, *MOGAT2*, *SRF*, *ATP5G2*, *TP53*, *ATP1B2*. For each gene, microarray (log2) signals are plotted on the x-axis and qPCR (2^−∆Ct^) data on the y-axis. Corresponding correlation coefficients (r) and *p-values* are shown.

**Figure 2:**
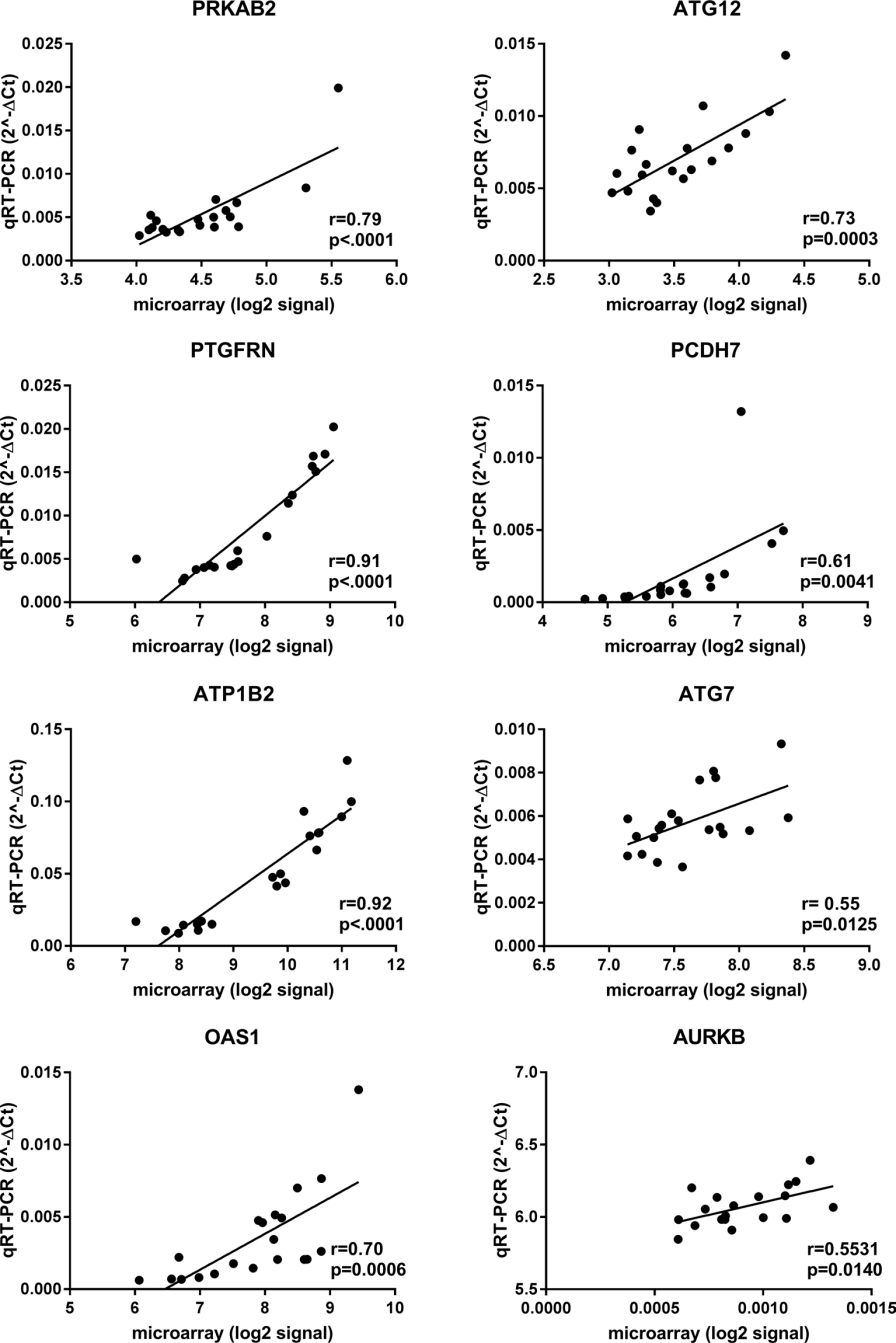
qPCR validation of selected genes of the adrenal gland microarray study, including *AURKB*, *PRKAB2*, *ATG12*, *PTGFRN*, *PCDH7*, *ATP1B2*, *ATG7*, *OAS1*. For each gene, microarray (log2) signals are plotted on the x-axis and qPCR (2^−∆Ct^) data on the y-axis. Corresponding correlation coefficients (r) and *p-values* are shown.

Real-time quantitative PCR (qPCR) validation: To cross-validate platform performance, a selection of microarray revealed DEGs were tested in gene-specific quantitative qPCR assays. Eight hypothalamic DEGs were selected for qPCR based on their functional role in one of the top canonical pathways suggested by IPA. Correlation analysis between microarray and qPCR of *AURKB*, *CCK*, *GNA12*, *MOGAT2*, *SRF*, *ATP5G2*, *TP53* and *ATP1B2* in the hypothalamus resulted in significant correlation coefficients ranging from 0.41 (*p* < 0.006) to 0.81 (*p* < 0.0001) (Fig. 1). Likewise, for the adrenal gland, the mRNA abundances of *AURKB*, *PRKAB2*, *ATG12*, *PTGFRN*, *PCDH7*, *ATP1B2*, *ATG7* and *OAS1* measured by the two methods were significantly correlated with a Pearson correlation coefficient ranging from 0.55 (*p* = 0.0125) to 0.92 (*p* < 0.0001) as shown in Fig. 2. Together, our data suggest a good concordance between the microarray and qPCR results.

## Discussion

Improving animal welfare and reducing the psychosocial stress of domestic pigs is a vital issue in modern pig production. Several studies explored coping behavior from various angles, focusing on behavioral^3^, physiological^23^ and immunological^24^ aspects. Krause *et*. *al* demonstrated coping style-dependent central physiological differences in autonomic nervous system regulation, which is controlled by the hypothalamus^25^. For our study, we selected animals based on the haplotype of candidate markers previously found to be highly associated with coping behavior^22^. These genomic regions, harboring several genes, exhibited high linkage disequilibrium (LD)^22^. The above mentioned haplotypes may be in close LD with the causal variant or may themselves consist of the causal variants of interest^26^. In addition, the biological functions of transcripts of specific tissues involved in the hypothalamic-pituitary-adrenal (HPA) axis from the same animals were taken into account. Coping behavior associated haplotype information in synergy with the evaluation of biological transcript function facilitates the discovery and broadens the knowledge of causal variants for complex phenotypes like coping behavior and thus might provide promising new biomarkers for genotype-based selection and animal welfare.

Significant differences between the HR and LR groups in all three backtest parameters (latency, duration, and frequency) emphasize an association of genetic based early-life coping style with differential mRNA expression in our study. Several previous transcriptomic studies focused on the differential mRNA expression associated with adrenal sensitivity to ACTH and psychosocial stress^27–30^. The present study is the first to report associations between genetics, temperament, and differential gene expression in porcine hypothalamus and adrenal gland tissues. Preslaughter handling including the use of electronarcosis to sacrifice the animals may affect animal welfare and stress response, particularly in brain regions. Animals of both groups were treated the same way. Plasma glucocorticoid levels are considered good markers of stress^31^, but in this study, the total plasma cortisol level measured by ELISA showed no significant difference between the HR and LR groups; although, it tended to be higher in the LR group. Our previous study measured cortisol levels in the absence of any backtest experiments in 475 individual pigs and showed levels of 93.9 ± 34.6 ng/ml (mean ± SD)^32^. The range of this baseline measurement lies between the cortisol measurement ranges of the HR and the LR group.

This observation suggested that the HR and LR pigs were likely not exposed to stressful conditions prior to sample collection and that the gene expression pattern was not directly influenced by the cortisol level. In this study the samples were taken at adult stage. A prior study has shown that repeated backtests reflect personality and coping strategy with a moderate intra-individual consistency and heritability^19^. More than 3000 animals, of which the animals used here represent a subset, it was previously shown that the correlations among the backtest at different time points (ages) (rs = |0.19–0.44|) strongly supports the backtest traits as reliable parameters of personality and coping style and for long life and inheritance^19^.

### Differentially-expressed transcripts in the hypothalamus

Due to its pivotal role in stress response, the transcriptomic signature of the hypothalamus is of particular interest and may shed light on the molecular basis of coping-related behavioral traits. Altered mRNA transcript abundances in the HR compared to LR group in the hypothalamus were associated with signaling of cholecystokinin (*CCK*), nerve growth factor (*NGF*), and their interaction as well as with oxidative phosphorylation, oxidative stress and mitochondrial dysfunction.

In the hypothalamus, we found that Cholecystokinin/Gastrin-mediated signaling is upregulated in the HR pigs. Besides its well-known role as a peptide hormone within the gastrointestinal system, cholecystokinin is found extensively throughout the central nervous system (CNS)^33,34^. Previous studies have reported a wide variety of behavioral and autonomic actions in different species, such as the suppression of exploratory behavior^35^, a modulatory role in memory formation and learning^36^, anti-opioid activity^37–39^, anxiety induction^40^, thermoregulation, and involvement with the dopaminergic reward system^41^. Human as well as rodent studies clearly suggest a positive causal relationship between CCK and anxiety^42^. A robust and dose-dependent release of ACTH and cortisol could be observed after intravenous injection of the CCK-B receptor agonist pentagastrin^43,44^. Preliminary data showed a relative resistance to cortisol feedback inhibition^43^. Consequently, CCK could be involved in an acute activation of the HPA axis, which is independent from the actual plasma cortisol level and in this way promotes its activity even under conditions of already elevated cortisol. It is noteworthy to mention that the anxiogenic and HPA effects of CCK are independent phenomena being coordinated by multiple pathways^44–46^. Beyond that and strikingly, *CCK-8* has been implicated in inhibiting oxidative stress-induced neurotoxicity via an anti-oxidative stress pathway^47^.

Higher basal gene expression of transcripts associated with the IPA biofunction *NGF* signaling in the HR group were observed in our study. As a highly conserved neurotrophin, nerve growth factor plays a pivotal role in the survival and maintenance of sympathetic and sensory neurons^48^. Interestingly, a relationship between *CCK* and *NGF* has been reported previously: intraperitoneally injected CCK-8 was shown to stimulate NGF synthesis via activation of CCK receptors^49^. Taking the role of CCK-8 regarding oxidative stress response into account, activation of CCK-/gastrin mediated signaling can be seen as one step in an oxidative stress-induced neuronal recovery process carried out by NGF. Animal and human studies report reduced body weight, increased anxiety, and mood changes after NGF administration^50,51^. Furthermore, NGF injection into the brains of cholinergic function- compromised rats induced hyperactivity and fear^49^.

The gene coding for the highly conserved transcription factor SRF presented with lower expression levels in the HR compared to LR pigs. Many immediate early genes such as *c-fos* are regulated by SRF. A regulatory function of SRF signaling is mediated by MICAL2, an atypical actin-regulatory protein^52^. In our study, *MICAL2* expression was elevated in the hypothalami but decreased in the adrenal glands of HR pigs compared to LR pigs. A basic capability of MICALs is the generation of redox potential, via their mono-oxygenase domains, and in turn reactive oxygen species (ROS)^53^. For this reason, we speculate that hypothalami of LR individuals face heightened ROS production due to increased *MICAL2* expression. Earlier studies have emphasized the role of *MICAL2* and several adjacent genes in mediating anxiety^54^.

*MUC1* was one of the top ten hypothalamic DEGs in our study (FC = −1.54, *p* < 0.0014). Its cytoplasmic tail is able to bind TP53, increasingly under conditions of genotoxic stress^55^. *TP53*, also one of the hypothalamic DEGs, aligns with QTL regions on SSC12 (55.2 Mb) and showed decreased transcript abundance in the HR pigs. Both molecules, *MUC1* und *TP53* are associated with the TP53 response element p21 gene promotor, leading to an activation of p21 and finally cell cycle arrest^55^. Additionally, *MUC1* expression increases as a cellular response to oxidative stress^56^; hence, its elevated expression in LR compared to HR pigs can be interpreted as an indicator of a derailed reactive oxygen species (ROS) balance, which may be caused by increased *MICAL2* expression in the LR pigs.

Due to their crucial role in cellular physiology, mitochondria are particularly sensitive to changes in cell homeostasis. It is therefore not surprising that we found different activation patterns of pathways related to oxidative phosphorylation and mitochondrial dysfunction in the LR and HR groups. DEGs associated with mitochondrial complexes I and III were lower in HR individuals. Even partial inhibition of complex I can cause enhanced ROS production, leading over time to oxidative stress and eventually neuronal damage^57^. Many psychiatric and neurological disorders are correlated with compromised mitochondrial function^58^, and increased anxiety-like behaviors have been observed in mice with mitochondrial dysfunction^59^. In a zebrafish study investigating chronic unpredictable stress (CSU)-induced anxiety, brain proteome profiling revealed differentially regulated proteins predominantly involved in mitochondrial function, glycolysis, oxidative stress, and the hypoxia stress response, emphasizing that mitochondrial dysfunction may be causal for the anxious phenotype^60^.

### Differentially-expressed transcripts in adrenal gland

As part of the HPA axis, the adrenal gland produces glucocorticoids which are able to affect almost any vertebrate cell by binding to the glucocorticoid receptor. Moreover the gland secretes catecholamines into the circulatory system in the course of sympathetic activation. These physiological roles make this tissue a promising target for elucidating the molecular pathways of coping behavior. The DEGs in adrenal gland have higher *q* values, despite using samples from the same animals we used for hypothalamic differential expression analysis. The adrenal gland constitutes the last component of the HPA axis which might explain the limited impact on differential gene expression between the HR and the LR pigs. Nevertheless, this organ is highly responsive to exogenous stressors and in fact, we found that the most significant adrenal signatures differing between the LR and the HR pigs were associated with glucocorticoid receptor signaling.

Cortisol and other glucocorticoids bind to the glucocorticoid receptor to execute gene regulatory functions in development, metabolism, and immune response. IPA associated 10 DEGs in our present study to the glucocorticoid receptor signaling pathway, including *BAG1*, *BCL2L1*, *CD247*, *ELK1*, *NFKBIA*, *POLR2G*, *POLR2H*, *SMAD2*, *STAT5B*, *TAF3* and *HDAC6*. Overall, activation of this pathway was reduced in HR compared to LR pigs. Downregulation of glucocorticoid receptor signaling ultimately entails downregulation of *NFKBIA* in the HR group. Expression of the *NFKBIA* gene, encoding for an inhibitor of NFKB-mediated inflammation, is stimulated by glucocorticoids^61,62^ and was shown to be strongly correlated with plasma cortisol concentrations^32^.

### Differentially expressed genes of both tissues in GWAS regions

Approximately 47% (17 out of 36 genes) of the DEGs in both tissues were located in GWAS regions which have been identified on SSC12^22^. These candidate genes in combination with their biological function will be selected for further analysis of the causal relationship of SNPs and behavioral traits. Results from the previous GWAS linking backtest parameters and coping style with significantly associated markers revealed a cluster of SNPs with LD in the region between 54 and 56 Mb of porcine chromosome SSC12 using the pig genome assembly Sscrofa10.2^22^. The corresponding region in the new pig genome assembly (Sscrofa11.1) is located between 52 and 54 Mb on SSC12. Among the tissue-wide DEGs in GWAS regions, three highly significant genes (FDR < 0.0001) located in the GWAS region on SSC12 deserve particular attention: *ATP1B2*, *MPDU1* and *AURKB*. *ATP1B2* exhibited significantly lower expression (FDR < 0.0001) in the LR group. By using qPCR for validation, we confirmed both differential expression of *ATP1B2* in the adrenal gland (*p* < 0.0001, FC = −4.04) and in the hypothalamus (*p* = 0.0175, FC = −1.54). Consistent with this, a study with two substrains of Wistar Kyoto rats (high mobility and low mobility), which were subjected to forced swim test, revealed differential expression of *ATP1B2* in their hippocampi^63^. *ATP1B2* (also known as *AMOG*) encodes the beta 2 subunit of Na^+^/K^+^-ATPase. This sodium pump is responsible for the electrochemical gradient of Na^+^ and K^+^ ions and thus maintains membrane potential and cellular homeostasis^64^. In addition, dysfunction of *AMOG* might influence the ionic and osmotic regulation, contributing to neuronal hyperexcitability^65^. *ATP1B2*^−/−^ mice showed rapidly worsening motor impairment and degeneration of neural cells in the central nervous system^66^. A recent study suggest that the neurohistopathological changes seen in spongy degeneration with cerebral ataxia (SDCA) in Malinois dogs might be caused by a SINE insertion into *ATP1B2*, leading to aberrant RNA splicing and reduced ATP1B2 protein expression^67^. Mannose-P-dolichol utilization defect 1 protein, encoded by the *MPDU1* gene, plays an integral role in the synthesis of glycosylphosphatidylinositols and lipid-linked oligosaccharides^68^. Homozygous point mutations in the *MPDU1* gene have been associated with congenital disorder of glycosylation type If in humans, a disease featured by, inter alia, psychomotor retardation and seizures^69^. The underlying cause of seizures is seen in abnormalities of neuronal activity in the brain due to excessive or synchronous firing^70^. The link between *MPDU1* and the occurrence of seizures in humans makes it tempting to speculate, that this gene participates in one way or another in neuronal activity regulation and that lower *MPDU1* expression could lead to abnormally excessive neuronal firing. *AURKB* encodes a member of the aurora kinase subfamily of serine/threonine kinases. Recently, it has been suggested that AurkB is involved in the regulation of neuronal development and axonal outgrowth^71^. Experiments, using pharmacological and genetic methods to impair AurkB function rendered motor axon morphology to a truncated and abnormal phenotype, whereas elongated axonal outgrowth has been observed after *AURKB* overexpression^71^. Both *AURKB* and *MPDU1* were found to be highly differentially expressed between HR and LR of both tissues but the functions of these genes with regard to coping behaviour still remain unknown. Another gene in this region, *NDEL1*, plays a role in multiple processes including cell signalling, neuronal migration and neurite outgrowth. Ndel1 is responsible for motor protein (dynein) regulation together with LIS1. Disruption of this complex leads to impaired neuronal migration by altering nuclear-centrosome coupling^72^. By its ability to bind DISC1, the NDEL1/Lis1 complex is involved in the development of the neurobehavioral symptoms of schizophrenia^73^. A genome-wide study investigating the genetic factors of schizophrenia found an association between the enzyme activity of NDEL1 and disease symptoms, with lower levels of NDEL1 activity in the case group^74^. A well described human neuronal migration disorder caused by aberrant Lis1/Ndel1 function is Miller-Dieker syndrome (MDS)^75^. Affected individuals suffer from severe mental retardation as well as epileptic seizures, suggesting that *NDEL1*, like *MPDU1*, might contribute to excessive neuronal firing. Located on SSC7, *FAH* belongs to the promising DEGs in QTL regions of both tissues. Fumarylacetoacetate hydrolase (FAH) catalyzes the last step of tyrosine degradation^76^, and tyrosine itself is the amino acid precursor of catecholamine synthesis^77^. The elevated mRNA transcript abundance of *FAH* in the LR group observed in our study suggests increased tyrosine degradation and thus an abated supply of catecholamine precursors with corresponding effects on the sympathetic activity of this group. In contrast, absence of *FAH* expression in mice brains produced a phenotype with markedly increased myelination and altered social behavior^78^.

In summary, transcriptomic profiling in combination with information on genetic variance and backtest behavior proved to be a versatile approach for understanding genotype-phenotype-mapping associated with targets of hypothalamic and adrenal gland gene expression and the physiological processes related to backtest behavioral traits. Our study demonstrated haplotype associated, coping pattern linked transcripts in the hypothalamus and the adrenal gland. These findings revealed meaningful biological pathways like oxidative phosphorylation, mitochondrial dysfunction, cholecystokinin/gastrin-mediated signaling, and NGF signaling – the latter two working hand in hand to counteract oxidative stress-induced neurodegenerative effects in the hypothalamus and themselves affecting behavioral traits. The glucocorticoid receptor pathway is plausible molecular pathway that plays a role in this context in the adrenal gland. 47% of the DEGs of both tissues were located in GWAS regions which have been identified on SSC12. Among them, *ATP1B2*, *MPDU1*, *AURKB* and *NDEL1* represent the most promising candidates for further analyses. The gene *ATP1B2* is involved in the maintenance of electrochemical gradients and neuronal excitability, *NDEL1* is associated with both neuronal migration and neurite outgrowth and despite clear evidence for a functional role in the nervous system; the exact link of *MPDU1* and *AURKB* to behavioral traits still needs to be unraveled. Altogether, the results of our study increase our understanding of causal variants for complex phenotypes like coping behavior in pigs. However, it is advisable to confirm the role of the identified genes and pathways for coping behavior in other pig populations and other ages of pigs, as alternative pathways may be relevant in other populations and ages. By narrowing down the list of candidate genes in GWAS regions, this study provides evidence for molecular correlates of coping behavior, which may have the welfare-enhancing potential to be used as biomarkers for animal wellbeing or genotype-based selection.

## Material and Methods

### Ethics statement

Animal care and tissue collection procedures followed the guidelines of the German Law of Animal Protection and the experimental protocol was approved by the Animal Care Committee of the Leibniz Institute for Farm Animal Biology as well as by the State Mecklenburg-Western Pomerania (Landesamt für Landwirtschaft, Lebensmittelsicherheit und Fischerei; LALLF M-V/TSD/7221.3-2.1-020/09). The experimental protocol was carried out in accordance with the approved guidelines for safeguarding good scientific practice at the institutions in the Leibniz Association and the measures were taken to minimize pain and discomfort and accord with the guidelines laid down by the European Communities Council Directive of 24 November 1986 (86/609/EEC).

#### Coping behavior and haplotype estimation

Animals were provided by the Leibniz Institute for Farm Animal Biology (FBN, Dummerstorf, Germany). 294 German Landrace pigs used in this study were subjected to backtests on days 5, 12, 19 and 25 *post natum* which was previously reported^22^. Briefly, each individual piglet was swiftly turned on its back and put onto a V-shaped device padded with cellulose. To avoid escape, the piglets were held manually in that position with slight pressure, adapted to the intensity of their movements. The test commenced immediately after reaching immobility and lasted for 60 s. Latency (L), the time elapsed until the first struggling attempt, duration, the cumulative struggling time (D) within the 60 s test, and frequency (F), the number of struggling attempts, were recorded. Moreover, total latency (tL), total duration (tD), and total frequency (tF) were calculated for each animal by summing up the relevant parameters at all ages tested.

Based on our previously reported of a prominent QTL region harboring a coping-behavior associated haplotype block on SSC12, we used the following 6 markers (ALGA0066975, ALGA0121951, ASGA0055092, ASGA0105202, H3GA0034753 and MARC0073387), located in a linkage disequilibrium (LD) block within the GWAS region of SSC12 (Supplementary Fig. S1), for haplotype estimation and trend regression analysis^22^. To estimate the unobserved haplotype frequencies, the haplotype estimation process in JMP Genomics (SAS Institute, Cary, NC, USA) was used. This process invokes an expectation-maximization (EM) algorithm to estimate haplotype frequencies. Estimates of haplotype frequencies were used as input for the Haplotype Trend Regression process, in order to further determine the particular haplotype from the 6 SNPs in LD regions. To test for association of each haplotype with backtest parameters, PROC GLIMMIX was used with sex as a fixed effect and sire as a random effect. In total, 4 haplotype variants of 294 piglets were used to test the association against backtest phenotype. The data set lists the F-statistics and associated probabilities for each of the estimated haplotypes. HR animals are homozygous carriers of haplotype 1 (A_G_A_C_A_A), whereas all LR animals are carriers of haplotype 2 (G_A_G_A_G_C). A total of 20 pigs with high-reactive (HR, n = 10) or low-reactive (LR, n = 10) coping patterns together with the information of the haplotype were selected from a pool of 294 German Landrace (DL) piglets.

### Sample collection

Pigs were weighed and slaughtered by electronarcosis followed by exsanguination. The average age of the 20 animals was 157 days *post natum*. Blood samples were collected in 50 ml Falcon tubes containing 1 ml of 0.5 M EDTA and immediately stored on ice. Subsequently, plasma sample separation was performed, and samples were kept at a temperature of −80 °C until use. An enzyme-linked immunosorbent assay (ELISA, DRG, Marburg, Germany) was carried out in duplicate as described previously^22^ in order to provide total plasma cortisol levels. Additionally, tissue samples for the determination of transcript abundances were taken immediately after exsanguination. For the dissection of hypothalamus, a stereotaxic atlas of the porcine brain was used as reference guide^79^. The whole hypothalamic area, including the paraventricular nucleus, was extracted surgically out of the left and right hemispheres after quickly harvesting the brains. In brief, the skull was opened with a saw. The optic chiasma and the nucleus mamillaris anterior (Mammilary body) up to the thalamus were removed. Laterally of the anterior commissure along the thalamus was cut. The collected tissue corresponded to the width of the mammilary body. This procedure has ensured that the Nucleus paraventricularis hypothalami and the Nucleus arcuatus hypothalami were part of the collected tissue. Middle portions of both adrenal glands were excised. After taking tissue from the hypothalamus and adrenal gland, the samples were snap frozen in liquid nitrogen and stored at −80 °C until use.

### RNA isolation target preparation and hybridization

Total RNA was isolated from hypothalamus and adrenal gland tissue samples using the TRI Reagent (Sigma-Aldrich, Taufkirchen, Germany) and RNeasy kit (Qiagen, Hilden, Germany). The RNA was quantified using the NanoDrop ND-1000 spectrophotometer (Peqlab, Erlangen, Germany), checked for integrity by performing agarose gel electrophoresis (1% agarose gel), and stored at −80 °C until use. To prepare the samples for microarray analysis, amplified sense-strand cDNA was generated using the Ambion WT Expression Kit (Ambion, Austin, TX, USA). Next, the cDNA was fragmented and biotin-labeled using the Affymetrix GeneChip WT Terminal Labeling Kit (Affymetrix, Santa Clara, CA, USA). Each individual sample was hybridized on a genome-wide snowball array (Affymetrix, Santa Clara, CA, USA), containing 47880 probe-sets. After a staining and washing step, the arrays were scanned and processed using Affymetrix GCOS 1.1.1 software.

### Microarray data processing

The data was pre-processed using Affymetrix Expression Console 1.3.1.187 software (Affymetrix), and the normalization was done using the RMA (robust multichip average) algorithm. The DABG (detection above background) algorithm was used to filter present (expressed) genes. Probes-sets present in less than 80% of the total number of samples and those representing miRNAs were excluded from further analysis. Differential gene expression analysis between the LR and HR group was assessed by running mixed model procedure in JMP Genomics 7.0 (SAS Institute, Cary, NC, USA) with sex and coping style as fixed effects and sire as a random effect. The adjusting for multiple comparisons across the Type 3 tests for all of the fixed effects was calculated using the *post hoc* Tukey-Kramer test. To control for multiple testing, *p*-values were converted to a set of *q*-values^80^. The level of significance was set at *p* < 0.05 which corresponds to *q* < 0.17 for hypothalamus and *q* < 0.47 for adrenal gland, respectively. Differentially expressed genes (DEGs) were used for pathway analysis using Ingenuity Pathway Analysis (IPA) (Ingenuity Systems, Redwood City, CA). IPA categorizes genes based on annotated gene functions and statistically tests for over-representation of functional terms within the gene list using Fisher’s exact test.

The identification of candidate genes with differential expression between the LR and the HR group from both tissues was implemented using the mixed model procedure in JMP Genomics 7.0 (SAS Institute, Cary, NC, USA) with sex, coping style and tissue as fixed effects and sire as a random effect. The model was combined with a *repeated* statement with animal as the subject effect, in order to take into account the correlations among measurements made on the same animal in both tissues. The *post hoc* Tukey–Kramer method was used for multiple comparison adjustments. To control for multiple testing, the FDR was set to 0.05^81^.

### Real-time quantitative PCR (qPCR) validation

DEGs were selected for qPCR validation based on their functional roles in the IPA significant pathways using a 48 × 48 Dynamic array with an integrated fluidic circuit (IFC) on the BioMark HD Real-time PCR System (Fluidigm, South San Francisco, CA, USA). cDNA was generated using 2 µg of total RNA, Superscript II reverse transcriptase (Invitrogen, Carlsbad, CA, USA), and oligo(dT), with specific target amplification (STA) and Exonuclease I treatment. A total of 2.25 µL of the sample treated with STA and Exo-I, 2.5 µL of SsoFast EvaGreen supermix with low ROX (Biorad, Hercules, CA, USA), and 0.25 µL DNA binding dye sample loading reagent were loaded for each sample inlet. Assay inlets were loaded with 2.25 µL DNA suspension buffer, 2.5 µL assay loading reagent, and 0.25 µL of a 100 µM primer solution (forward and reverse). Temperature scheme for the qPCR reaction was as follows: An initial denaturation step for 60 s at 95 °C, followed by 30 cycles consisting of denaturation at 95 °C with 5 s each and annealing at 60 °C for 20 s. Reference genes (*HPRT1* and *RPS11*) were selected based on microarray data as invariant genes between LR and HR group. All measurements were performed in duplicate. Primer sequences can be accessed in Supplementary Table S3.

## Supplementary information

Supplementary Figure 1

Supplementary Table 1, Supplementary Table 2, Supplementary Table 3

## Data Availability

The data used in this study was deposited in the database of the National Center for Biotechnology Information Gene Expression Omnibus (www.ncbi.nlm.nih.gov/geo) [GEO: GSE109155, GSM293170-2933189 and GSM2933190-2933209].
