# Supplementary material for: Transcriptome profiles of hypothalamus and adrenal gland linked to haplotype related to coping behavior in pigs
Source: Sci Rep. 2019 Sep 10;9:13038. doi: 10.1038/s41598-019-49521-2 (PMC6736951; doi:10.1038/s41598-019-49521-2)
Supplement: Supplementary file 1 — Supplementary Figure 1 [file 41598_2019_49521_MOESM1_ESM.pdf]

Transcriptome profiles of hypothalamus and adrenal gland linked to haplotype related to coping behavior in pigs

Kevin Gley1, Eduard Murani1, Nares Trakooljul1, Manuela Zebunke2,3, Birger Puppe3, Klaus Wimmers1, Siriluck Ponsuksili1*

FigureS1: Linkage disequilibrium (LD) of single nucleotide polymorphisms (SNP) located between 54 and 56 Mb on Sus scrofa chromosome (SSC) 12 that were significantly associated with backtest results; modified after Ponsuksili et al.; 2015; Sci Rep.; 5: 16264.

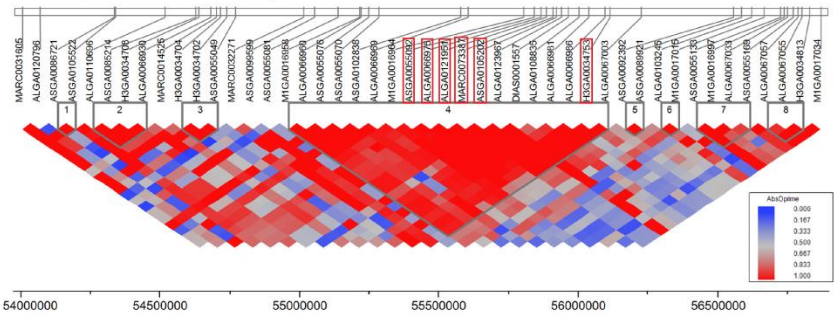

Level of LD between loci indicated by color as depicted in the key. LD block 4 between 55 and 56 Mb was the largest block, harboring the six markers used for calculation of haplotype association (depicted with a red frame

Supplementary Figure 1.

Linkage disequilibrium of SNPs located between 54 and 56 Mb on SSC12 that were significantly associated with backtest traits.
